# Morphology Evolution in High‐Performance Polymer Solar Cells Processed from Nonhalogenated Solvent

**DOI:** 10.1002/advs.201500095

**Published:** 2015-05-26

**Authors:** Wanzhu Cai, Peng Liu, Yaocheng Jin, Qifan Xue, Feng Liu, Thomas P. Russell, Fei Huang, Hin‐Lap Yip, Yong Cao

**Affiliations:** ^1^ Institute of Polymer Optoelectronic Materials and Devices, State Key Laboratory of Luminescent Materials and DevicesSouth China University of TechnologyGuangzhou510640P. R. China; ^2^ Materials Science DivisionLawrence Berkeley National LabBerkeleyCA94720USA; ^3^ Polymer Science and Engineering DepartmentUniversity of MassachusettsAmherstMA01003USA

**Keywords:** additives, high‐performance solar cells, morphology evolution, nonhalogenated solvents, polymer solar cells

## Abstract

**A new processing protocol based on non‐halogenated solvent and additive** is developed to produce polymer solar cells with power conversion efficiencies better than those processed from commonly used halogenated solvent‐additive pair. Morphology studies show that good performance correlates with a finely distributed nanomorphology with a well‐defined polymer fibril network structure, which leads to balanced charge transport in device operation.

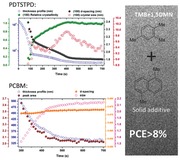

Polymer solar cells (PSC), featuring high mechanical flexibility, light‐weight, and low manufacturing costs, have reached a milestone in performance with power conversion efficiency (PCE) over 10% and are considered an important renewable energy source.^1^, ^2^, ^3^, ^4^, ^5^ One of the critical issues that determine PSCs' performance is the morphology of the bulk heterojunction (BHJ) blends. Typically, a high‐performance PSC system will require a multiple‐length scale of phase separation between the donor and the acceptor, which guarantees large specific inner surface area to effectively split excitons and provide bicontinuous pathways for efficient charge carrier transport.^6^ Choice of processing solvent plays an important role in regulating the morphology of blended thin films, since the solvent not only provides adequate solubility for both the donor and the acceptor but also affects the interactions between them^7^, ^8^ and can modulate the crystallinity of the polymer donors.^9^, ^10^

At present, most of the best performing PSCs were processed from chlorinated solvents, such as chlorobenzene (CB) and 1,2‐dichlorobenzene (DCB), with small amounts of processing additives, such as 1,8‐diiodooctane (DIO), 1,8‐octanedithiol (OT), or 1‐chloronaphthalene (CN) to achieve the optimal morphologies and PCEs. In general, aromatic chlorinated solvents are good solvents for both conjugated polymers and C_60_ derivatives.^11^, ^12^ Additives with high boiling points and selective solubilities can dramatically influence the size scale of the phase separation of the components.^13^, ^14^, ^15^ By processing with chlorinated solvents, well‐organized polymers and PCBM domains can be obtained^9^, ^16^ and even a vertical gradual‐component‐distributed morphology can be constructed,^17^, ^18^ which are beneficial to the carrier transport and extraction.

While chlorinated solvents are nearly ideal for controlling the BHJ active layer morphology, it is impractical to use these hazardous solvents for large‐scale manufacturing and the development of more environmental‐friendly processes based on nonhalogenated solvents is critically important.^19^, ^20^, ^21^, ^22^, ^23^ Ideally, water‐based processing could be used, but designing water‐soluble organic semiconductors to generate high PCE devices is a challenge that is yet to be met.^24^, ^25^ Nonhalogenated aromatic solvents and additives have also been explored to produce PSCs with encouraging results and showed PCEs comparable to those processed from chlorinated solvents.^26^, ^27^ A particularly interesting solvent system is based on methylbenzenes as the host solvent and naphthalene derivatives as the additive solvent. Methylbenzenes typically show good solubility for conjugated polymer donors but not for the fullerene acceptors. Therefore, small amounts of naphthalene derivatives, a kind of very good solvent for fullerenes,^12^ are required to prevent severe aggregation of fullerenes during the film drying process and achieve the desired nanoscale phase‐separated morphologies. Even though these solvent systems showed general applicability to many PSC materials, their effect on the formation of the BHJ morphology, a balance of multiple kinetic processes, is poorly understood. Consequently, establishing a better understanding of the structure–performance relationship for PSC based on these solvents is important before advancing to large‐scale production of PSCs.

Here, we report a new processing protocol based on a nonhalogenated host solvent and additive for producing PSCs with a performance outperformed that from systems using CB and DIO, a commonly used solvent–additive pair. The morphology/performance relationship was thoroughly studied using atomic force microscopy (AFM), transmission electron microscopy (TEM), resonance soft X‐ray scattering (RSoXS), and in situ grazing incidence X‐ray diffraction (GIXD). The donor polymer used in this study is a well‐studied copolymer of dithienosilole and thienopyrrole‐4,6‐dione (PDTSTPD), which showed a high PCE (up to 7%) when blended with PC_71_BM.^28^, ^29^, ^30^ 1,2,4‐trimethylbenzene (TMB) was chosen as the host solvent as it is a good solvent for the donor polymer and 1,5‐dimethylnaphthalene (1,5‐DMN) was chosen as the processing additive as it interacts strongly with PCBM.^12^ Optimized PDTSTPD:PC_71_BM‐based PSCs processed from this new solvent system showed PCEs over 8%, much better than devices prepared from CB:DIO, pure TMB, and pure CB solvents. The thin film morphology and structure evolution of the morphology were further investigated using in situ GIXD study, which provided insights into the roles of each solvent during the solvent evaporation.

The donor polymer PDTSTPD is a low bandgap copolymer with a push‐pull structure, which has an thieno[3,4‐c]pyrrole‐4,6‐dione (TPD) as the electron‐deficient unit and dithienosilole (DTS) unit as the electron‐rich unit (see **Figure** **1**a).^30^ PDTSTPD offers a low‐lying highest occupied molecular orbital (HOMO) level, which yields a high open‐circle voltage (*V*_oc_) up to ≈0.9 V.^31^ The TPD reduces the bandgap down to 1.73 eV, which is beneficial for light harvesting. Besides, the interaction of sulfur atom from thienyl and the oxygen atom from imide, combining the alkyl chains interaction in the TPD unit, promotes molecular planarity and lamellae formation, resulting in high order molecular packing in the solid state.^28^, ^32^ For device fabrication, a PDTSTPD:PC_71_BM ratio of 1:2 was used, and the active layer thickness was ≈ 90 nm. Four processing solvents (solutions) were used: TMB, TMB with 30 mg mL^−1^ 1,5‐DMN, CB, CB with 3 vol% DIO. The chemical structures of each are shown in Figure 1a.

**Figure 1:**
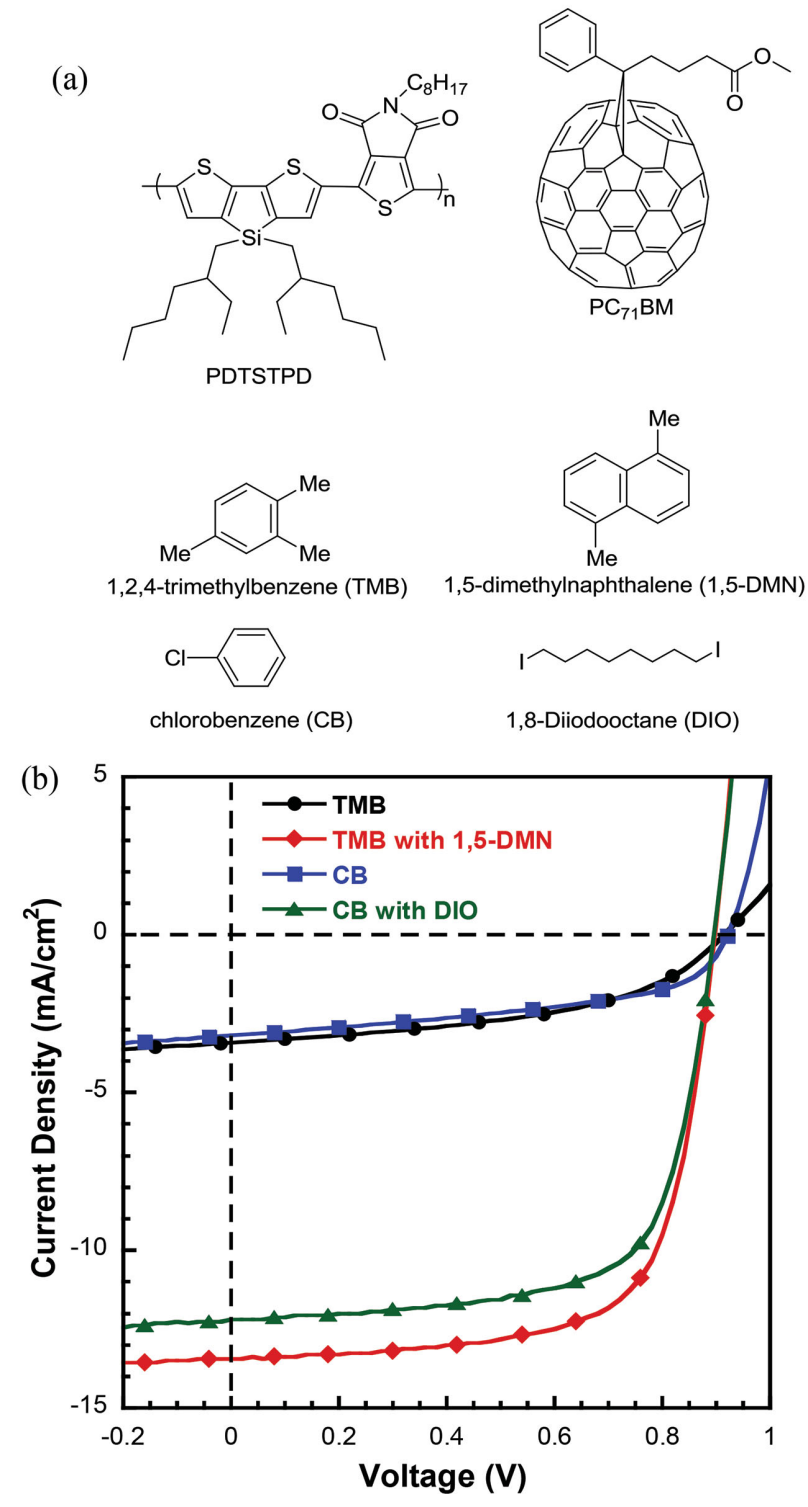
a) Chemical structure of PDTSTPD, PC_71_BM, host solvent molecule (TMB and CB), and additive (1,5‐DMN and DIO). b) Current–voltage (*J*–*V*) curves of devices processed by different solvents under simulated 100 mW cm^−2^ AM 1.5 G illumination, device configuration is ITO/PFNOX/Active layer/MoO_3_/Al, the active area of the device is 0.16 cm^2^.

An inverted device structure of ITO/PFNOX/Active layer/MoO_3_/Al was used, where PFNOX is an interfacial layer designed to modify the work function of the ITO cathode, ensuring efficient electron collection.^33^ The *J*−*V* curves of the devices under simulated 100 mW cm^−2^ AM 1.5 G illumination are shown in Figure 1b. The reference device, processed from CB with DIO, had a PCE of 7.48%, a *V*_oc_ of 0.90 V, a short circuit current density (*J*_sc_) of 12.21 mA cm^−2^, and a fill factor (FF) of 68.1%, very similar to that reported previously.^30^ In the nonhalogenated cases, the PCE of TMB+1,5‐DMN devices increased to above 8%, with a *J*_sc_ of 13.42 mA cm^−2^, an FF of ≈70%, and a *V*_oc_ of 0.90 V. These results exceed the highest PCE for PDTSTPD‐based devices, arising primarily from an increase in the *J*_sc_. For a comparison, 1,2‐DMN, which had been used as an additive in the previous report,^27^ was also tested and the corresponding devices showed performance slightly lower than that processed with 1,5‐DMN. The performance and morphology of devices processed from TMB+1,2‐DMN are summarized in Figure S2 (Supporting Information). The PCEs of devices processed from pure TMB and CB showed PCEs of 1.40% and 1.37%, respectively. All the performance para­meters are summarized in **Table** **1**. In order to further evaluate the solvent effect in different types of device structures, devices with conventional structure were also fabricated and we found that the trend observed is the same as that in the inverted cells, suggesting that the strategy can be widely adopted for different device architectures. The *J*–*V* curves and performance data for the conventional cells are provided in Figure S1 and Table S1 in the Supporting Information.

**Table 1:** Photovoltaic performance of polymer solar cells based on PDTSTPD:PC_71_BM processed from different solvents. (The standard deviation was calculated from measured results of six devices for each condition)

Solvents	*V*_oc_ [V]	*J*_sc_ [mA cm^−2^]	FF [%]	PCE [%]
TMB	0.89 ± 0.02	3.42 ± 0.19	45.9 ± 3.0	1.40 ± 0.12
TMB+1,5‐DMN	0.90 ± 0.01	13.54 ± 0.27	66.8 ± 1.8	8.10 ± 0.26
CB	0.92 ± 0.00	3.10 ± 0.12	48.0 ± 1.7	1.37 ± 0.10
CB + DIO	0.91 ± 0.01	12.34 ± 0.49	65.4 ± 4.2	7.32 ± 0.13

The increase in *J*_sc_ found in TMB+1,5‐DMN processed inverted device is a direct consequence of enhanced external quantum efficiency (EQE) when compared to CB + DIO device (**Figure** **2**a). Devices processed from pure TMB and CB showed much lower EQEs, <20%. For devices processed with additive, the enhancement of the EQE was mainly contributed from the polymer absorption. Thus in either 1,5‐DMN or DIO processing condition, light extraction of conjugated polymer is better fulfilled. TMB+1,5‐DMN processed devices showed an increase in broad spectrum in EQE, indicating a better morphology for charge generation and transport. Shown in Figure 2b are the absorbance spectra of blend films. Two strong peaks for PDTSTPD at ≈614 and ≈675 nm were observed (Figure S3, Supporting Information). The former arises from an internal charge transfer (ICT) between the DTS and TPD units, while the latter resulted from the vibronic interaction of intermolecular stacking.^30^, ^34^ Absorption spectra of the additive‐processed films are strongly overlapped in the PDTSTPD absorption band. For them, the intensity ratio of 675 nm peak to 614 nm peak obviously increased, suggesting a possible enhancement in polymer chain stacking.

**Figure 2:**
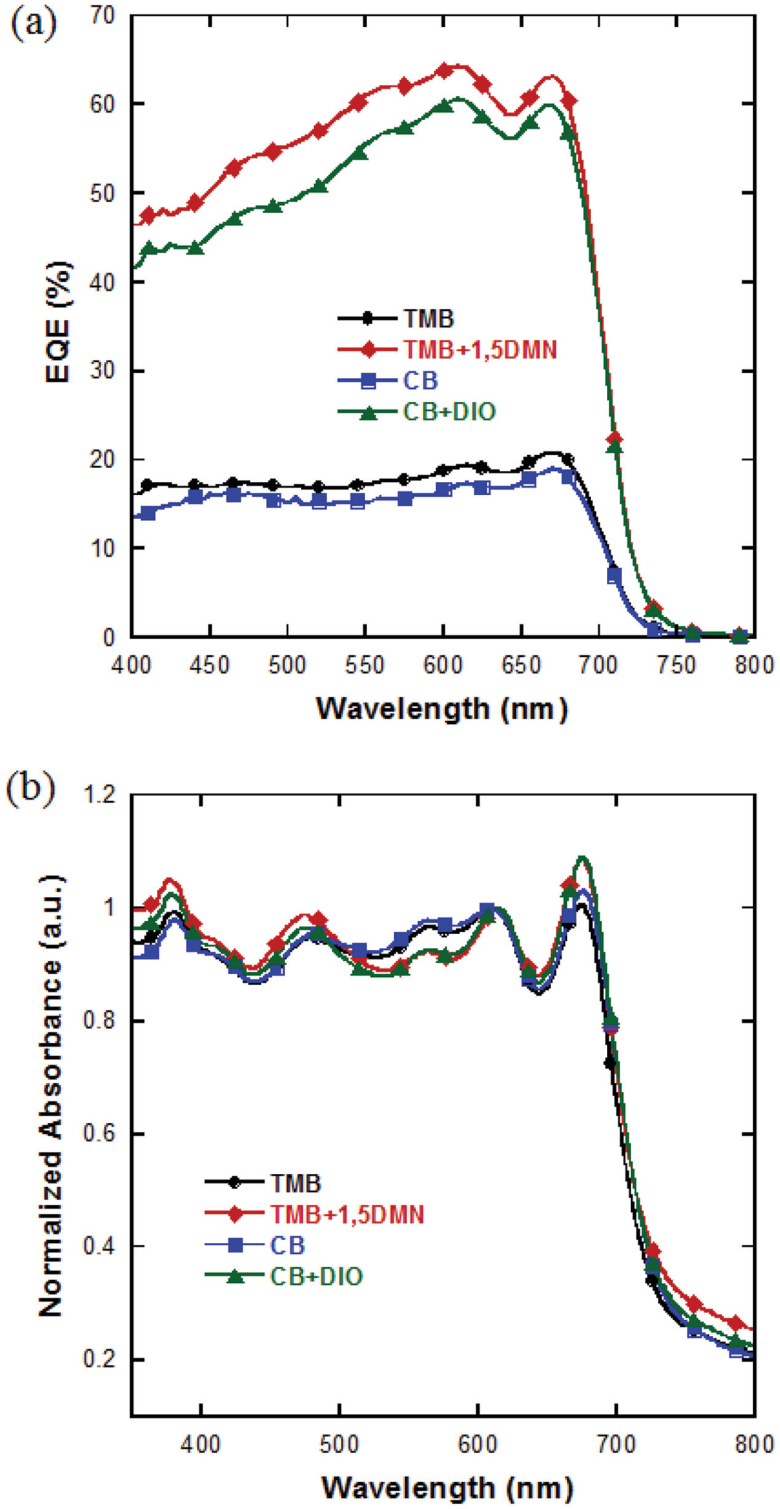
a) EQE spectra of the devices and b) normalized absorbance of blend films processed from different solvents.

To determine the effect of different additives on carrier transport, the hole and electron mobilities were evaluated from the *J–V* characteristic of single carrier devices. The hole‐only device structure was Al/MoO_3_/Active layer/PEDOT:PSS/ITO, and the electron‐only device was ITO/PFNOX/Active layer/PFN/Al. The mobilities were deduced by fitting the dark *J*–*V* curves to a Pool–Frenkel law modified space‐charge‐limited‐current (SCLC) model.^35^ The *J*–*V* curves, as well as the fitting results, are summarized in the Supporting Information (Figures S4, S5, and Table S2). For TMB devices, the hole and electron mobilities were both ≈10^−6^ m^1/2^ V^−1/2^, which is low but the two are balanced. When 1,5‐DMN was used as the additive, the hole mobility of devices increased two orders of magnitude to ≈10^−4^ m^1/2^ V^−1/2^, and the electron mobility also increases to a comparable level. This improved carrier transport agrees well with device current and EQE results. In contrast, for a CB device, the electron mobility (≈10^−5^ m^1/2^ V^−1/2^) was higher than the hole mobility (≈10^−6^ m^1/2^ V^−1/2^) by one order of magnitude, which is commonly observed in PSC system such as P3HT^36^ or PTB7^14^ processed from a non‐optimized processing solvent. This imbalance in carrier mobilities resulted in a deterioration in the performance.^37^ When the additive of DIO was used to process the device, the hole mobility was also dramatically increased by two orders of magnitudes (≈10^−4^ m^1/2^ V^−1/2^), while the electron mobility increased less significantly to a similar level.

GIXD was used to assess the crystallinity of PDTSTPD. Shown in **Figure** **3**a,b, CB and CB+DIO processed thin films showed a wide azimuthal distribution of (100) diffraction peak, which is narrower in TMB and TMB+1,5‐DMN cases. The (100) peak located at 0.31 Å^−1^, corresponding to a spacing of 2.0 nm. The π–π stacking peak is located at 1.69 Å^−1^, corresponding to a distance of 0.37 nm. In blends, the π–π stacking peak was located between the two PC_71_BM diffraction rings (1.3 and 1.9 Å^−1^), making it difficult to quantify. The (100) diffraction peak in the CB case showed a full width at half maximum (FWHM) of 0.13035 Å^−1^ (corresponding to a crystal size of 4.8 nm). The FWHM deceased to 0.07888 Å^−1^ (corresponding to a crystal size of 8.0 nm) in the CB+DIO case. With TMB, a FWHM of 0.1213 Å^−1^ (corresponding to a crystal size of 5.2 nm) was found which increased to 0.1508 Å^−1^ (corresponding to a crystal size of 4.2 nm) in TMB+1,5‐DMN processing. Thus 1,5‐DMN and DIO additives play a quite different role in modulating the crystal behavior of PDTSTPD in morphology evolution, which most probably comes from their different chemical content and interactions with conjugated polymers. DIO is an alkyl chain additive, which can favorably interact with side chains of PDTSTPD; 1,5‐DMN is aromatic additive, which can be favorably interact with backbone.

**Figure 3:**
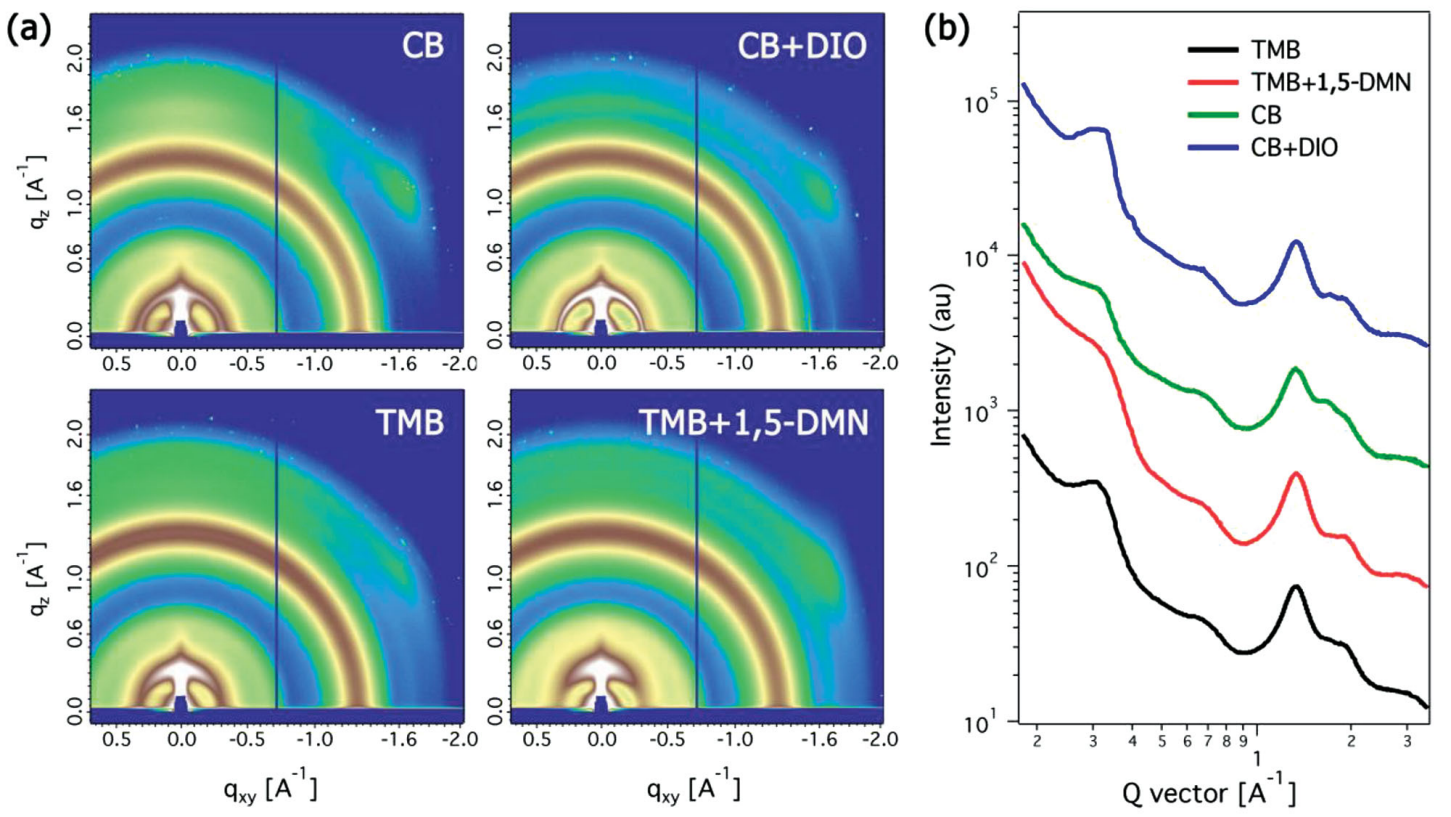
a) GIXD image and b) line‐cut profiles of GIXD along the out‐of‐plane direction of blend films processed from different solvents.

The global morphology of those blends was characterized by transmission electron microscopy (TEM). Shown in **Figure** **4**a, for the TMB film, dark irregularly shaped structures with a size up to ≈500 nm were observed, which are attributed to PC_71_BM‐rich domains.^38^, ^39^ For the CB film, PC_71_BM‐rich domains with size scales from ≈200 to ≈400 nm were also observed. These domains are far larger than the exciton diffusion length of ≈20 nm,^40^ which are disadvantageous for the exciton to split. The low electron mobilities observed in both the films processed from TMB and CB could be explained by the poor percolated pathways of the PC_71_BM‐rich domains. The inefficient exciton splitting and charge transport in these films led to poor *J*_sc_ and *FF* and accounted for the low PCEs. For the TMB+1,5‐DMN film, the material components appeared to be uniformly intermixed and large domains were not found. This observation was consistent with the AFM images (Figure S7, Supporting Information), which showed an RMS roughness of 1.88 nm compared to 8.34 nm in the film processed from pristine TMB. As a result, the number of interfaces for splitting the excitons was dramatically increased, and the carrier transport pathways formed by the PDTSTPD and PC_71_BM were finely distributed throughout the film and therefore led to a significantly increase in photocurrent. In the TEM image of CB+DIO‐processed film, the formation of large domains was suppressed but clusters of polymer crystals were observed. The less uniform distribution of the polymer crystals and less optimized percolation made the hole mobility in CB+DIO case slightly lower than that of TMB+1,5‐DMN‐processed films and this also explained the slightly lower in FF for the case of CB+DIO‐processed devices.

**Figure 4:**
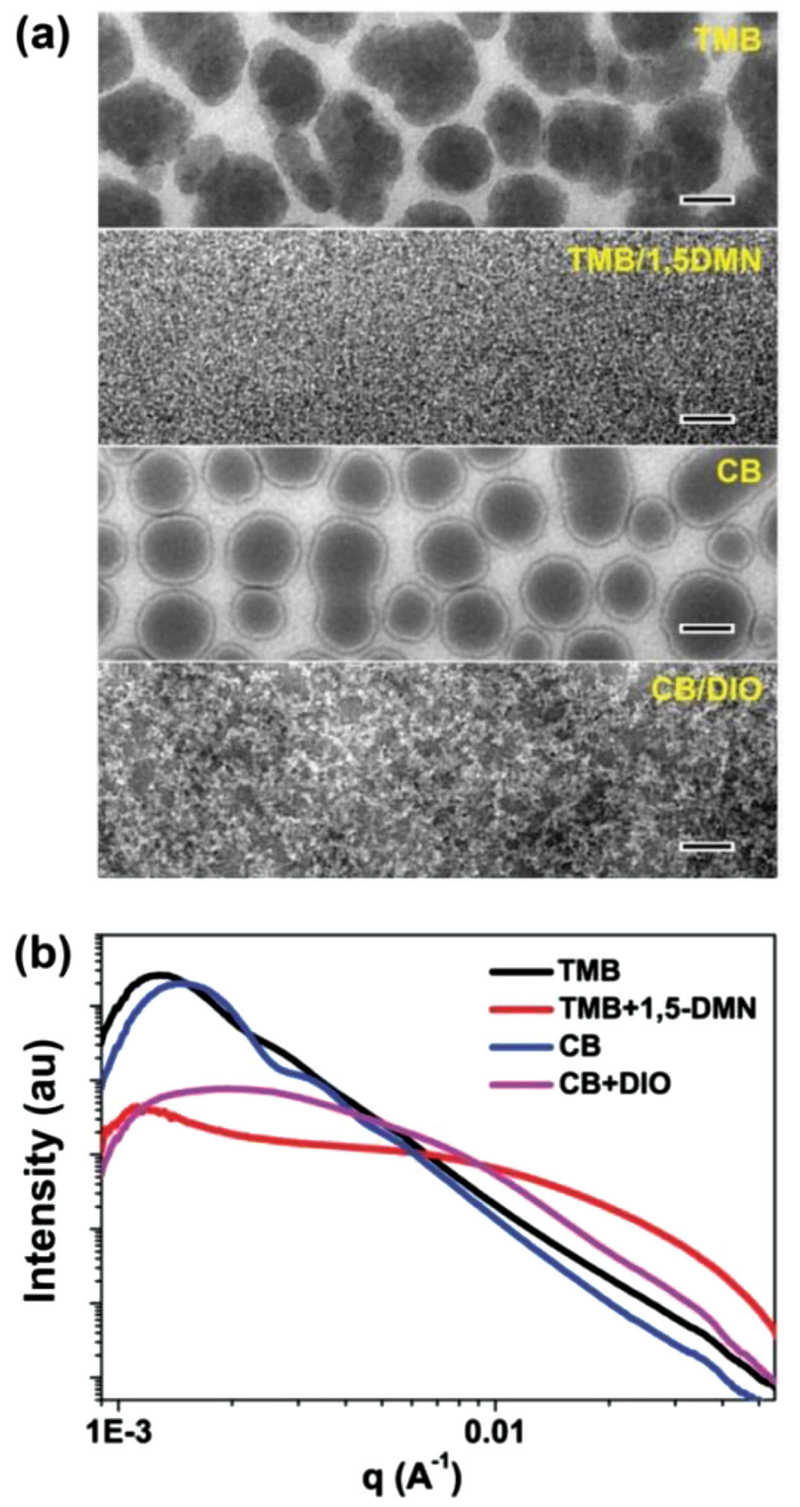
a) TEM images and b) RSoXS profiles of PDTSTPD:PC_71_BM (1:2 wt, ratio) blend films processed by different solvents, scale bar is 200 nm.

Resonant soft X‐ray scattering (RSoXS) study was performed to provide a better statistical average of the phase‐separated morphology. The scattering profiles of the corresponding films were plotted as a function of the scattering vector *q* in Figure 4b. The profile for the TMB‐processed film showed a strong reflection at 0.0013 Å^−1^, corresponding to an interdomain distance of ≈483 nm. For the CB‐processed film, a peak at 0.0015 Å^−1^, corresponding to a spacing of ≈419 nm, was observed when the additive was introduced into the processing, the scattering profiles changed dramatically. The TMB+1,5‐DMN profile showed a slow decay from low *q* to high *q*, and featured with a peak in the low‐*q* region (0.0012 Å^−1^, most probably coming from film thickness variation) and a broad, diffuse hump at ≈0.02 Å^−1^, corresponding to a distance of ≈31 nm. This length scale is the feature size of the donor–acceptor phase separation, in agreement with that observed by TEM image (the fibril‐to‐fibril spacing). Though broad in distribution, this length scale is much smaller in comparison to the TMB‐processed film and is consistent with a fourfold increase in photocurrent. The CB+DIO profile showed a peak located at 0.002 Å^−1^ and a broad hump in the high‐*q* region. Observations in RSoXS agree well with the multilength scaled morphology seen in the TEM image. The large scaled phase separation is ascribed to interspacing of polymer crystal clusters and smaller size scaled phase separation is the interfibrils spacing in a cluster, which gives rise to the elevated current in device operation. The existing small length scale phase separation is close to, but still larger than, that seen in the TMB+1,5‐DMN‐processed film. Consequently, a similar current of 12.21 mA cm^−2^ is obtained.

To provide a more in‐depth understanding on the morphology formation process during the solvent drying of the BHJ film processed from TMB+1,5‐DMN, the kinetics of the morphology evolution was therefore studied using in situ GIXD.^9^, ^41^ PDTSTPD:PC_71_BM blend was blade coated onto PEDOT:PSS coated wafer and a synchrotron X‐ray beam was used to monitor the crystallization of PDTSTPD during solvent evaporation. **Figure** **5**a shows the GIXD profiles obtained during solvent evaporation. The diffraction from 0.9 to 2 Å^−1^ arises from the solvent. It is evident that the PDTSTPD was fully dissolved. With time and as the solvent evaporated, the (100) peak at ≈0.3 Å^−1^ and PC_71_BM peak at ≈1.3 Å^−1^ became more prominent. Data analysis is summarized in Figure 5b,c. Before discussing the evolution process, we divided the drying period into three regions according to the different thickness changing, which are indicated by the dashed black line marked in Figure 5. In region I, the thickness of blend film decreases rapidly, due to rapid evaporation of TMB. In region II, the reduction in the thickness was slow as the residual host solvent was mostly gone and the additive evaporation process began to dominate the process. In region III, the thickness of the film is stabilized. In the beginning of region I, no polymer crystallization was seen. And clear polymer (100) peak appeared after ≈80 s, which quickly grew from 80 to 110 s (beginning of region II), as indicated by the increase in the (100) intensity (relative crystallinity) and the decrease in the *d*‐spacing. In region II, the crystal size of the PDTSTPD continues to increase, with an overall increase in the intensity (relative crystallinity), indicated an ordering of the PDTSTPD. The reduction in the *d*‐spacing, from 2.2 to 2.0 nm, indicates ordering of the PDTSTPD crystals is pushing out residual solvent or additive molecules trapped between the chains. The crystal size was stable in the first half of region II and then began to increase. In region III, the relative crystallinity and the *d*‐spacing of the PDTSTPD are stable, but the crystal size decreased. This, more than likely, arises from the removal of trapped solvents or additive molecules, leading to a disruption of the crystals. The PC_71_BM peak analysis is summarized in Figure 5c. Since the PC_71_BM peak and solvent peak were in the same region, it was difficult to quantify the results prior to removal of most of the solvent. Therefore, our peak fitting began from 300 s, when the majority of solvent and additives had evaporated. It is seen in Figure 5c that the 1.3 Å^−1^ peak areas follow the same trend with film thickness evolution, indicating the extent of PC_71_BM aggregation did not increase in the late stages of solvent evaporation, though a steady‐state increase in aggregate size was seen in region II, which stabilized in region III.

**Figure 5:**
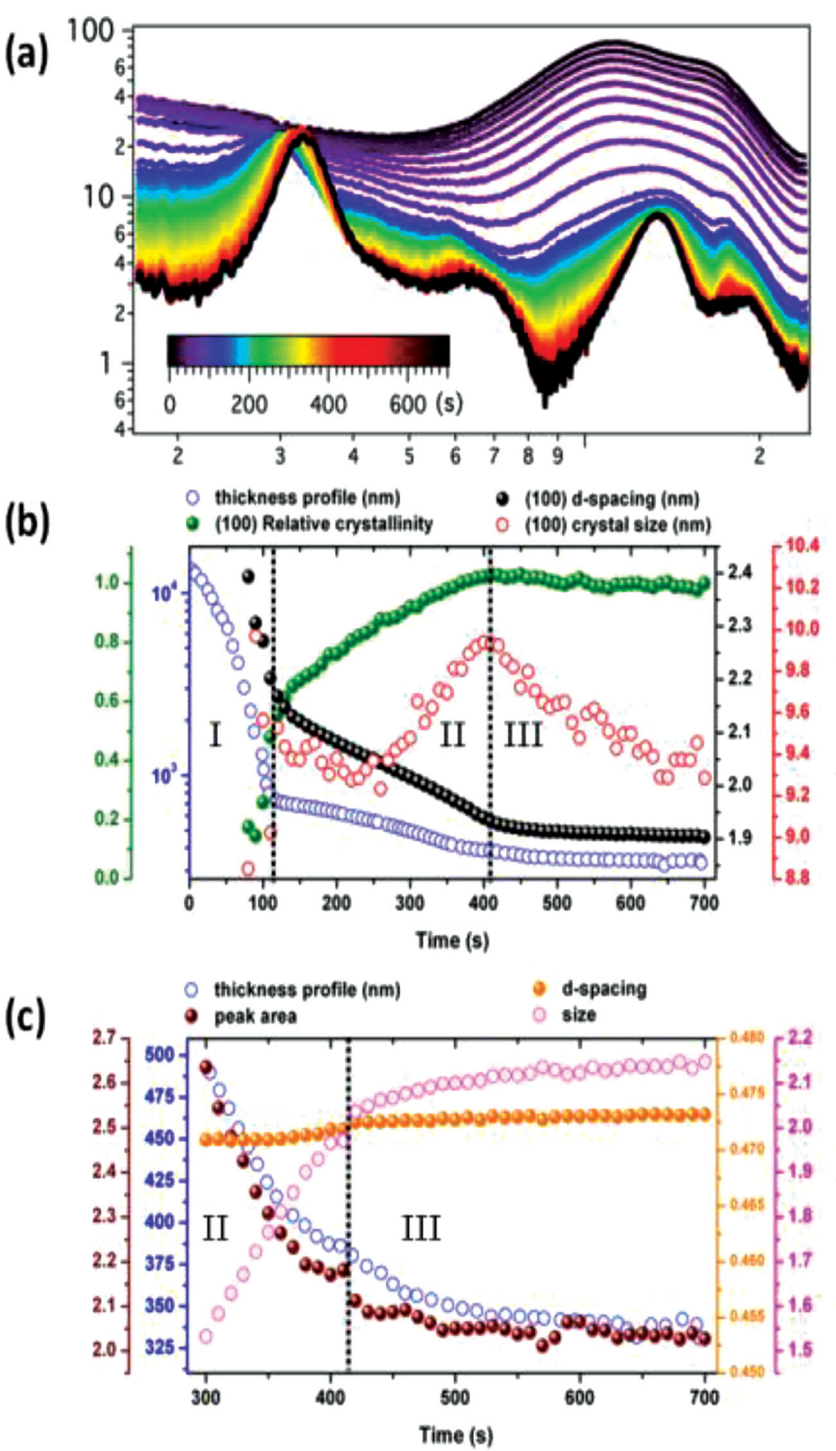
a) In situ GIXD and b) the data overlay for the (100) deflection peak of PDTSTPD and c) for PC_71_BM peak from the PDTSTPD:PC_71_BM film processed from TMB+1,5‐DMN with steady solvent evaporation.

To the aim of developing nonhalogenated‐solvent‐processed PSCs, devices based on PDTSTPD:PC_71_BM achieved PCEs over 8% when a small amount of solid additive 1,5‐DMN was introduced into the host processing solvent TMB. This processing method is less hazardous and delivers a performance higher than the conventional chlorinated solvent processing, pointing out new directions in PSC research. Morphology studies showed that good performance correlated with a finely distributed nanomorphology with a well defined polymer fibril network structure, which led to balanced charge transport in device operation. In situ GIXD experiments showed that the additive played a critical role in polymer crystallization and morphology evolution. Although polymer started to order in the later state of solvent evaporation, the major ordering occurred during the removal of the additive, which occurred over a longer time period, allowing the polymer chains longer time to order. The findings of this work not only demonstrated the potential of PDTSTPD but also the potential to use nonchlorinated solvents and additives in PSC processing in large‐scale device fabrication.

## Experimental Section

*Materials*: Monomer DTS was purchased from Suna Tech, Inc. Monomer TPD was synthesized and characterized according to the procedures reported in ref. 42. 
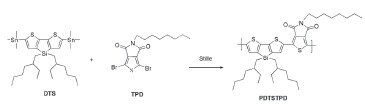


*Synthesis of Polymer PDTSTPD*: 4,4‐bis(2‐ethylhexyl)‐2,6‐bis(trimethyltin)‐dithieno[3,2‐*b*:2′,3′‐*d*]silole (DTS) (197.4 mg, 0.265 mmol) and 1,3‐dibromo‐5‐octylthieno[3,4‐*c*]pyrrole‐4,6‐dione (TPD) (107 mg, 0.253 mmol) were added to a flask under argon with 5.5 mL of degassed toluene/*N*,*N*‐dimethylformamide (DMF) (10:1,v/v). The flask was purged with argon for 15 min. The catalyst tetrakis(triphenylphosphine)palladium(0) (Pd(PPh3)4) (23 mg, 8%) was added quickly under a stream of argon, and then the reaction mixture was purged with argon again for 15 min. Subsequently, the resulting solution was stirred at 115 °C under argon. After 42 h of polymerization, 2‐(tributylstannyl)thiophene (80 μL) in 0.75 mL of degassed toluene/DMF (10:1, v/v) was added to the reaction flask and the reaction was kept at 115 °C for an additional 3.5 h. Bromobenzene (100 μL) in 0.75 mL of degassed toluene/DMF (10:1, v/v) was then added to the reaction flask, and the temperature was kept at 115 °C for an additional 7 h to complete the end‐capping reaction. The dark‐blue polymerization solution was cooled to room temperature and precipitated in methanol. The resultant polymer was collected by filtration, dried, and extracted successively with hexane and dichloromethane using a Soxhlet extraction apparatus. The remaining solid was extracted with 200 mL of chloroform. After concentration of the chloroform solution under reduced pressure, it was precipitated in methanol and the polymer, with a metal luster, was collected by filtration.

*PDTSTPD*: Yield (131 mg, 76%); Mn = 17 kDa and PDI = 2.11; 1H NMR (300 MHz, CDCl3): *δ* (ppm) = 8.45 (s, 1H), 7.39 (s, 1H), 3.69 (br, 2H), 2.1–0.15 (m, 49H).

*Devices*: Inverted device architecture was adopted in this study, ITO/PFNOX/PDTSTPD: PC_71_BM/MoO_3_/Al, in which ITO was the anode and exposed to light. The ITO‐coated glass substrates were cleaned by successive sonication in acetone, detergent, deionized water, and isopropyl alcohol. Then, the substrates were put into a baking oven under 80 °C for overnight. The subsequent procedure was carried out in a nitrogen‐filled glove box. About 5 nm thickness of PFNOX was obtained by spin‐coating from methanol solution at 2000 rpm for 20 s and cross‐linked through baking under 150 °C for 20 min. The PDTSTPD:PC_71_BM (weight ratio: 1/2) layer (≈90 nm) was spin‐coated at 1000 rpm from CB or CB with 3 vol% DIO, at 900 rpm from TMB or TMB with 30 mg mL^−1^ 1,5‐DMN. PDTSTPD was fully dissolved in TMB‐based solvent under 80 °C. Then, the substrates were pumped down to high vacuum (4 × 10^−6^ mbar). MoO_3_ film (≈10 nm) was thermally evaporated onto active layer at a rate of 0.1 Å S^−1^. After that, the substrates were transferred to another vacuum chamber for aluminum deposition (2 × 10^−6^ mbar). Aluminum (≈100 nm) was thermally evaporated onto the active layer using a shadow mask, which defined the active area of the devices to be 0.16 cm^2^. Finally, the devices were encapsulated before performance measurement.

*Carrier‐Only Devices*: ≈40 nm PEDOT:PSS (Baytron P VP AI 4083) was spin‐coated onto O_2_‐plasma‐treated ITO substrates at 2500 rpm for 40 s and baked at 145 °C for 20 min in air. The processing condition of active layer was identical to those of solar cells. MoO_3_ film was thermally evaporated onto active layer at a rate of 0.1 Å S^−1^ under a pressure of 4 × 10^−6^ mbar. PFN was dissolved in methanol with a concentration of 0.5 mg mL^−1^ and 0.5 vol% acetic acid.

*Measurements and Characterization*: The *J*–*V* characteristics of photovoltaic devices in light were measured under ambient using a Keithley 2400 source‐measurement unit. The simulated solar light was provided by a 150 W xenon lamp with an AM 1.5 g filter, which is a commercial product (XES‐40S1) of SAN‐EI, Inc. The light intensity of this solar simulator was calibrated using a reference cell with a KG5 color filter which was purchased from PV Measurements, Inc. The *J*–*V* characteristics of photovoltaic devices and carrier‐only devices in dark were measured in a glove box using a Keithley 236 source‐measurement unit.

External quantum efficiency (EQE) measurements were taken using a monochromator (Newport, Cornerstone 130) joined to the same xenon lamp and a lock‐in amplifier (Stanford Research Systems, SR 830) coupled to a light chopper.

The mobilities were determined by fitting the *J*–*V* curve of carrier‐only devices to the model of space charge‐limited conduction with Poole–Frenkel field‐dependent mobility,^43^ which is described as
(1)
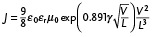
where *J* is the current density, *μ*_0_ is the zero‐field mobility, *ε*_0_ is the permittivity of free space, *ε*_r_ is the relative permittivity of the material, *γ* is the field activation factor, *L* is the thickness of the active layer, and *V* is the effective voltage. The effective voltage was achieved by subtracting the built‐in voltage (*V*_bi_) and the voltage dropped on series resistance (*V*_rs_) from applied voltage. In our measurement, the holes were injected from MoO_3_ side because of its better injection than PEDOT:PSS. *V*_bi_ is 0.1 V. The substrate's series resistance is ≈10 Ω. *ε*_r_ was always assumed to be 3, which is a typical value for organic materials. And the *V*_bi_ for electron‐only device is 0 V.

## Supporting information

As a service to our authors and readers, this journal provides supporting information supplied by the authors. Such materials are peer reviewed and may be re‐organized for online delivery, but are not copy‐edited or typeset. Technical support issues arising from supporting information (other than missing files) should be addressed to the authors.

Supplementary
