# Supplementary material for: Morphology Evolution in High‐Performance Polymer Solar Cells Processed from Nonhalogenated Solvent
Source: Adv Sci (Weinh). 2015 May 26;2(8):1500095. doi: 10.1002/advs.201500095 (PMC5115419; doi:10.1002/advs.201500095)
Supplement: Supplementary file 1 — Supplementary [file ADVS-2-0g-s001.pdf]

Copyright WILEY-VCH Verlag GmbH & Co. KGaA, 69469 Weinheim, Germany, 2015.

ADVANCED SCIENCE
Open Access

Supporting Information

for *Adv. Sci.*, DOI: 10.1002/advs.201500095

Morphology Evolution in High-Performance Polymer Solar Cells Processed from Nonhalogenated Solvent

Wanzhu Cai, Peng Liu, Yaocheng Jin, Qifan Xue, Feng Liu, Thomas P. Russell,* Fei Huang,* Hin-Lap Yip,* and Yong Cao*

Copyright WILEY-VCH Verlag GmbH & Co. KGaA, 69469 Weinheim, Germany, 2013.

Supporting Information

Morphology Evolution in High Performance Polymer Solar Cells Processed from Non-halogenated Solvent

Wanzhu Cai, Peng Liu, Yaocheng Jin, Qifan Xue, Feng Liu, Thomas P. Russell,* Fei Huang,* Hin-Lap Yip,* and Yong Cao*

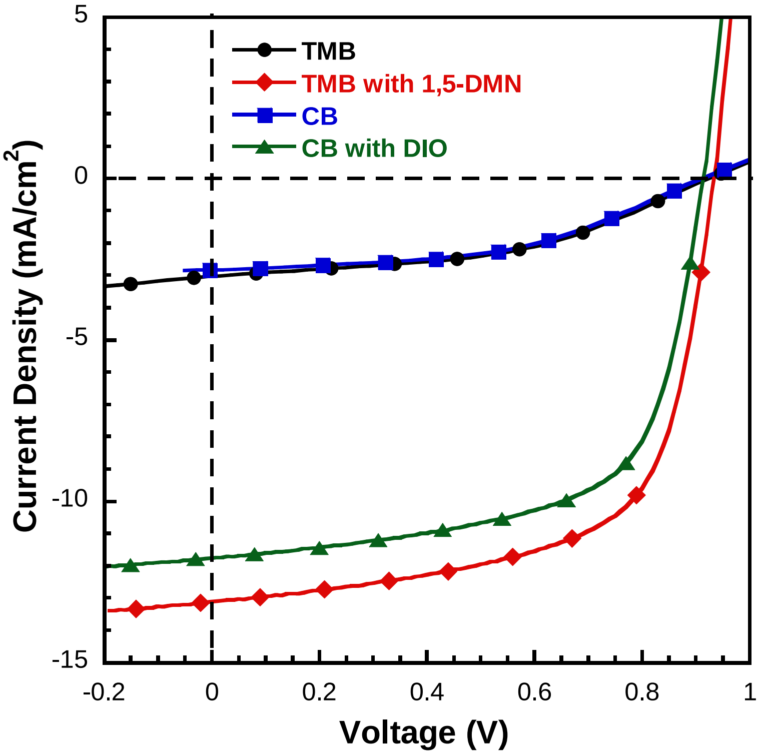

Figure S1. J-V curves of devices processed by different solvent under simulated 100 mW/cm^2 AM 1.5G illumination, device configuration is ITO/PEDOT:PSS/Active layer/PFN/Al, the active area of the device is $0.16cm^2$.

Table S1. Photovoltaic performance of polymer solar cells based on PDTSTPD: $PC_{71}BM$ processed from different solvent. Device configuration is ITO/PEDOT:PSS/Active layer/PFN/Al, the active area of the device is 0.16cm^2.

Solvents	V_{oc}(V)	J_{sc}(mA/cm^2)	FF(%)	PCE(%)
TMB	0.90±0.01	2.85±0.15	42.1±2.0	1.12±0.10
TMB+1,5-DMN	0.91±0.01	12.89±0.20	62.9±1.2	7.56±0.21
CB	0.89±0.01	2.71±0.10	45.3±1.4	1.11±0.09
CB + DIO	0.91±0.00	11.21±0.38	60.6±2.5	6.71±0.12

We have also studied the solvent additive effect of 1,2-dimethylnaphthalene, the improvement gain from 1,2-DMN is less than 1,5-DMN. And the results showed the phase separation of TMB+1,5DMN film is more pronounced.

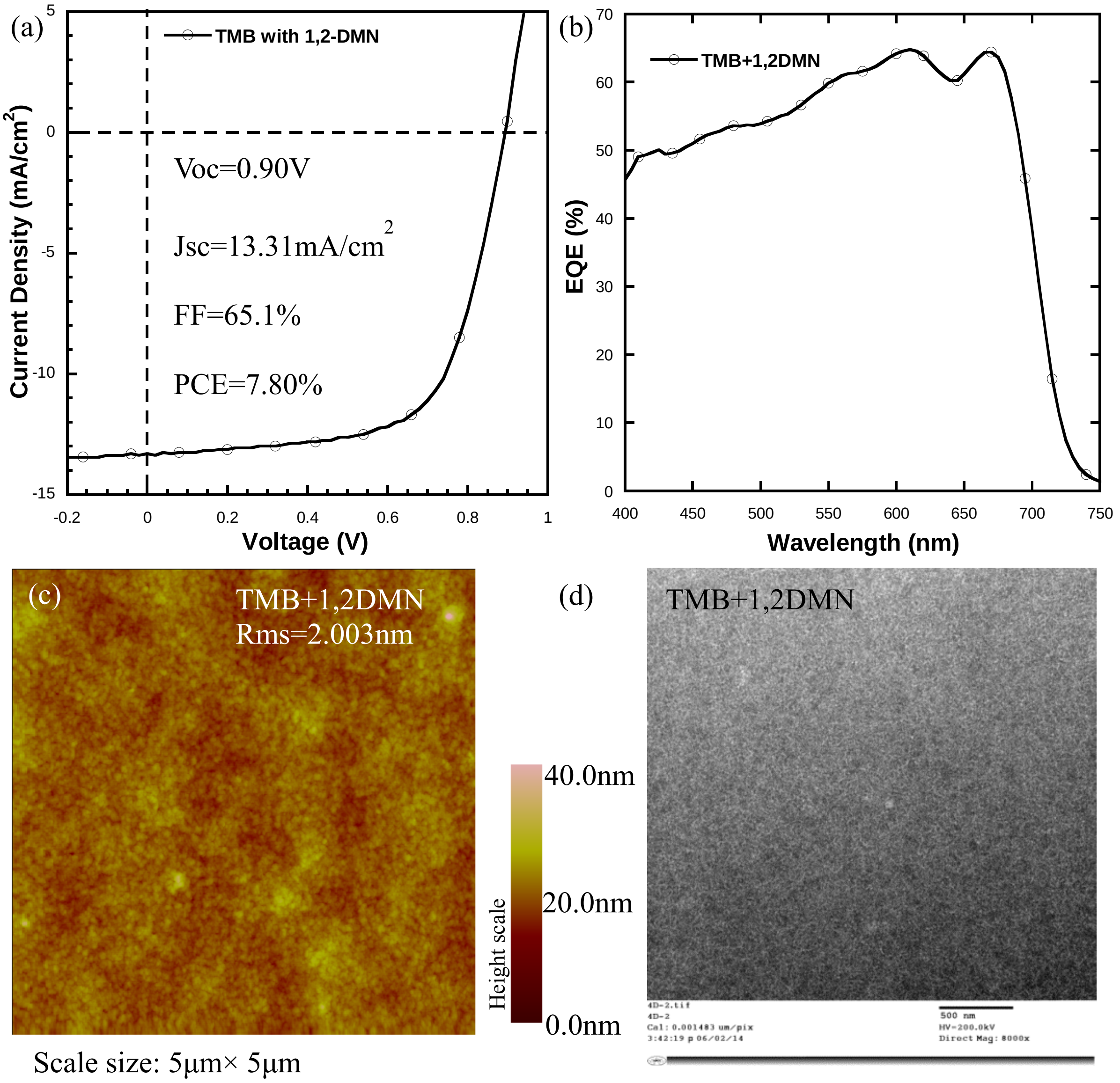

Figure S2. (a) J-V curves and (b) EQE spectra of devices processed by TMB with 1,2-dimethylnaphthalene under simulated 100 mW/cm^2 AM 1.5G illumination, device configuration is ITO/PFNOX/Active layer/MoO_3/Al. (c) AFM images and (b) TEM images of the corresponding film.

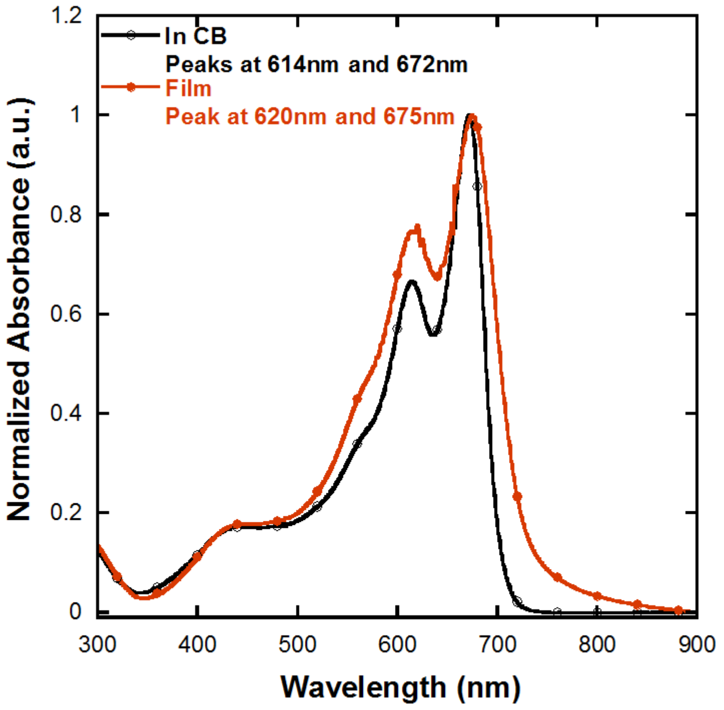

#

Figure S3. Normalized UV-Vis absorption spectra of pristine PDTSTPD in solution (black open circle) and films (red filled circle)#

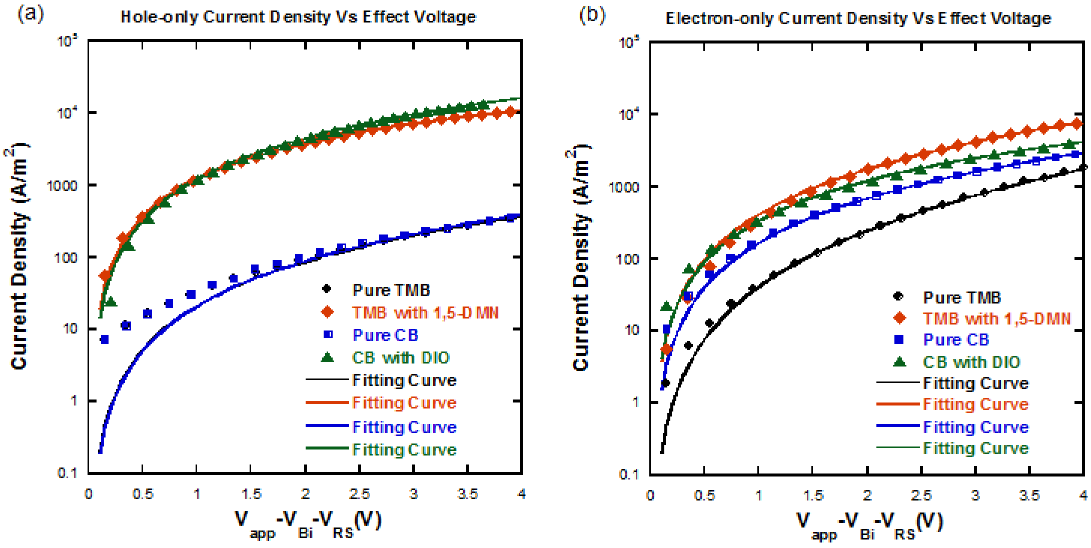

#

Figure S4. (a) J-V characteristics of devices in hole-only device ITO/ MoO_3/PDTSTPD:$PC_{71}BM$/ PEDOT:PSS /Al and (b) electron-only device ITO/PFNOX/PDTSTPD:$PC_{71}BM$/PFN/Al, in which the active layer was processed by different solvent. The solid lines represent the fitting curves.

Table S2. Fitting results contained mobility and field activation factor.

Solvent and Additive	Hole Mobility (cm^2/V/s), field activation factor (m$^{1/2}$ V$^{-1/2}$)	Electron Mobility (cm^2/V/s), field activation factor(m$^{1/2}$ V$^{-1/2}$)
Pure TMB	4.8×10^{-6}, 2.921×10^{-5}	3.7×10^{-6}, 3.39×10^{-4}
TMB with 1,5-DMN	5.8×10^{-4}, -2.117×10^{-4}	8.6×10^{-5}, -5.699×10^{-5}
Pure CB	4.4×10^{-6},5.32×10^{-5}	3.6×10^{-5}, 3.933×10^{-5}
CB with DIO	3.8×10^{-4}, -7.097×10^{-5}	1.1×10^{-4}, -8.575×10^{-5}

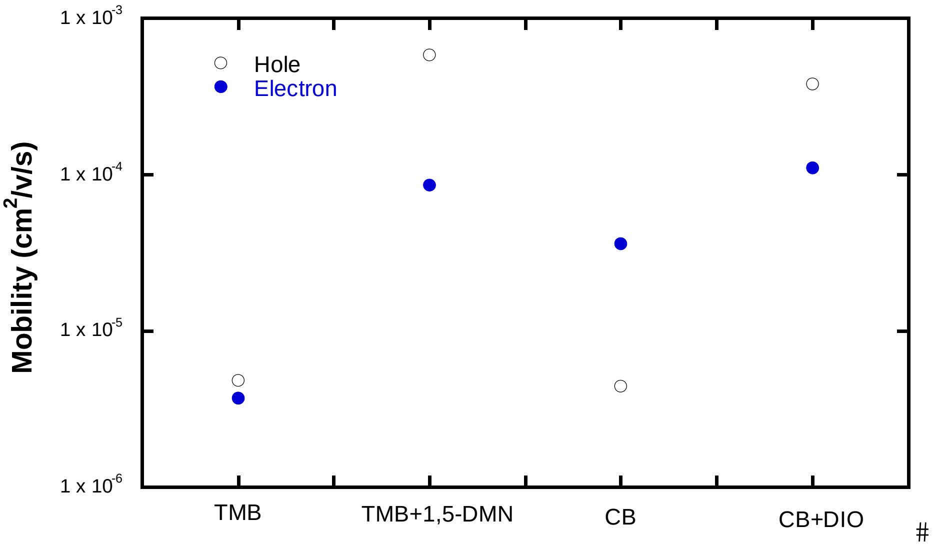

Figure S5. Mobility derived by SCLC model from J-V_{eff} characteristics of hole/electron only device.

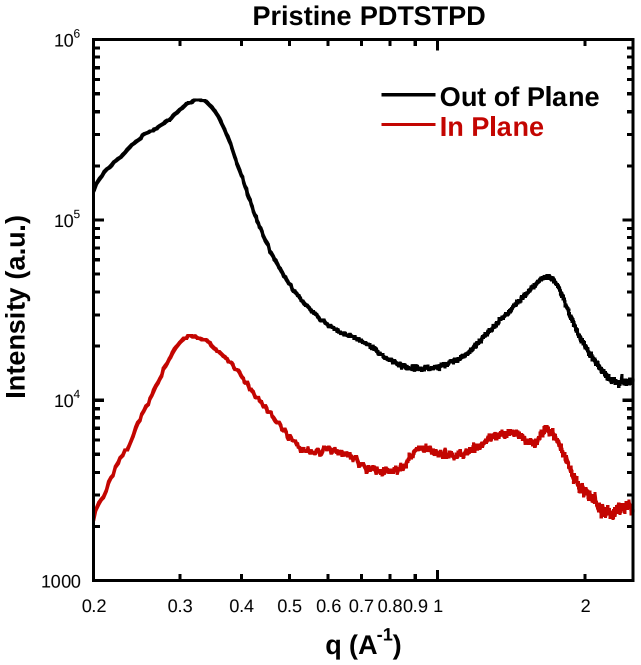

Figure S6. Line-cut profiles of GIXD of Pristine PDTSTPD film.

Figure S6 displays the GIXD profiles along the out-of-plane and in-plane direction of the 2D images for pure PDTSTPD film. In the out-off-plane direction, one strong (100) reflection at 0.31Å^{-1} along with a weaker (200) reflection at about 0.62Å^{-1} is observed at low q region, which attributes the interchain packing units with a space distance of 2.0 nm. The next peak at 1.69 Å^{-1} is from the (010) π-π* stacking plane reflection, corresponding to a space distance of 0.37 nm. In the in-plane direction, clear reflections for (100) reflection and 1.68 Å^{-1} for (010) reflection are also found, along with (200) and (300) reflection. These results suggest PDTSTPD molecule prefer a random crystalline orientation in pristine film.

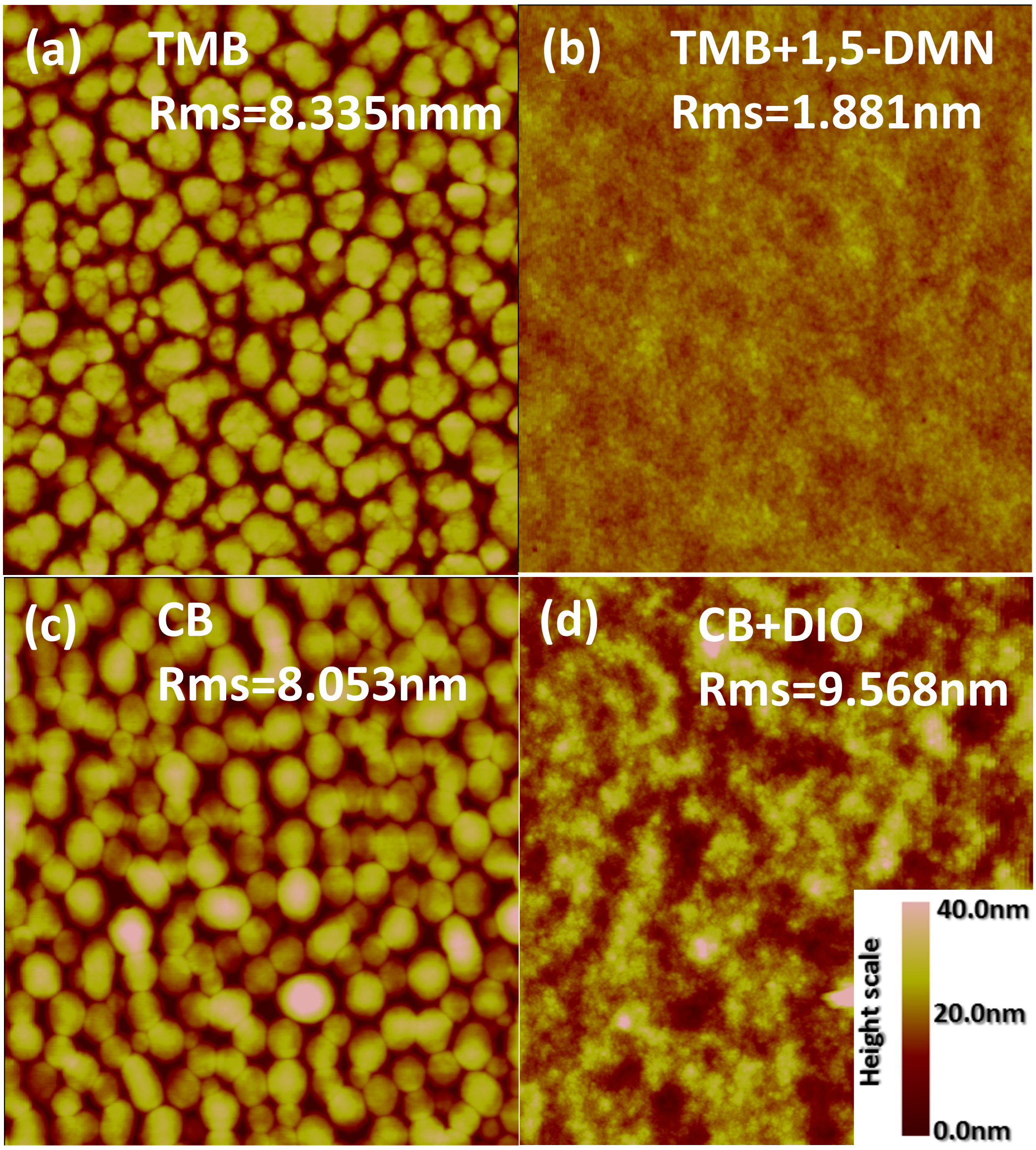

Figure S7. AFM images (5μm× 5μm) of PDTSTPD: $PC_{71}BM$ (weight ratio:1/2) blends films processed by different solvent. (a) Pure TMB, (b) TMB with 30mg/ml 1,5-DMN,(c) Pure CB, (d) CB with 3vol% DIO.

Table S3. Comparing the measured Jsc and theoretical Jsc of test cells.

Solvent and Additive	Measured Jsc(mA/cm2)	Calculated Jsc(mA/cm2)
Pure CB	3.10±0.12	3.3
CB with DIO	12.34±0.49	10.75
Pure TMB	3.42±0.19	3.63
TMB with 1,5-DMN	13.54±0.27	11.64
